# Supplementary figures and images for: Surviving on the edge: present and future effects of climate warming on the common frog (Rana temporaria) population in the Montseny massif (NE Iberia)
Source: PeerJ. 2023 Jan 13;11:e14527. doi: 10.7717/peerj.14527 (PMC9841900; doi:10.7717/peerj.14527)

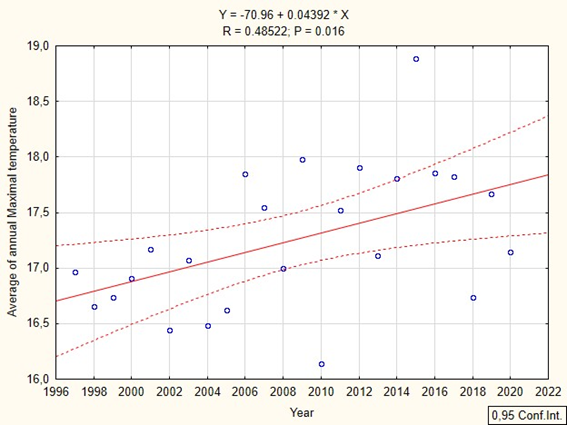

Supplement: Supplemental Information 1 [file peerj-11-14527-s001.png]

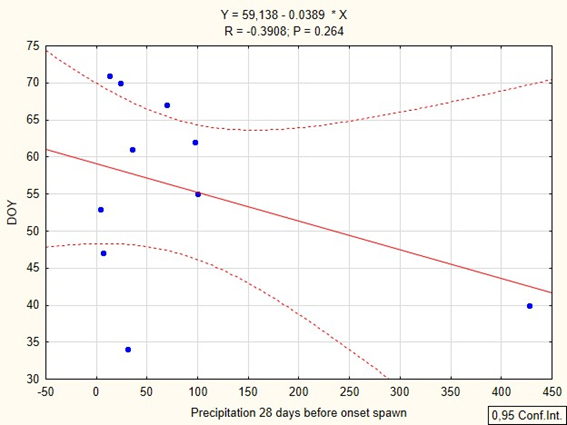

Supplement: Supplemental Information 2 [file peerj-11-14527-s002.png]

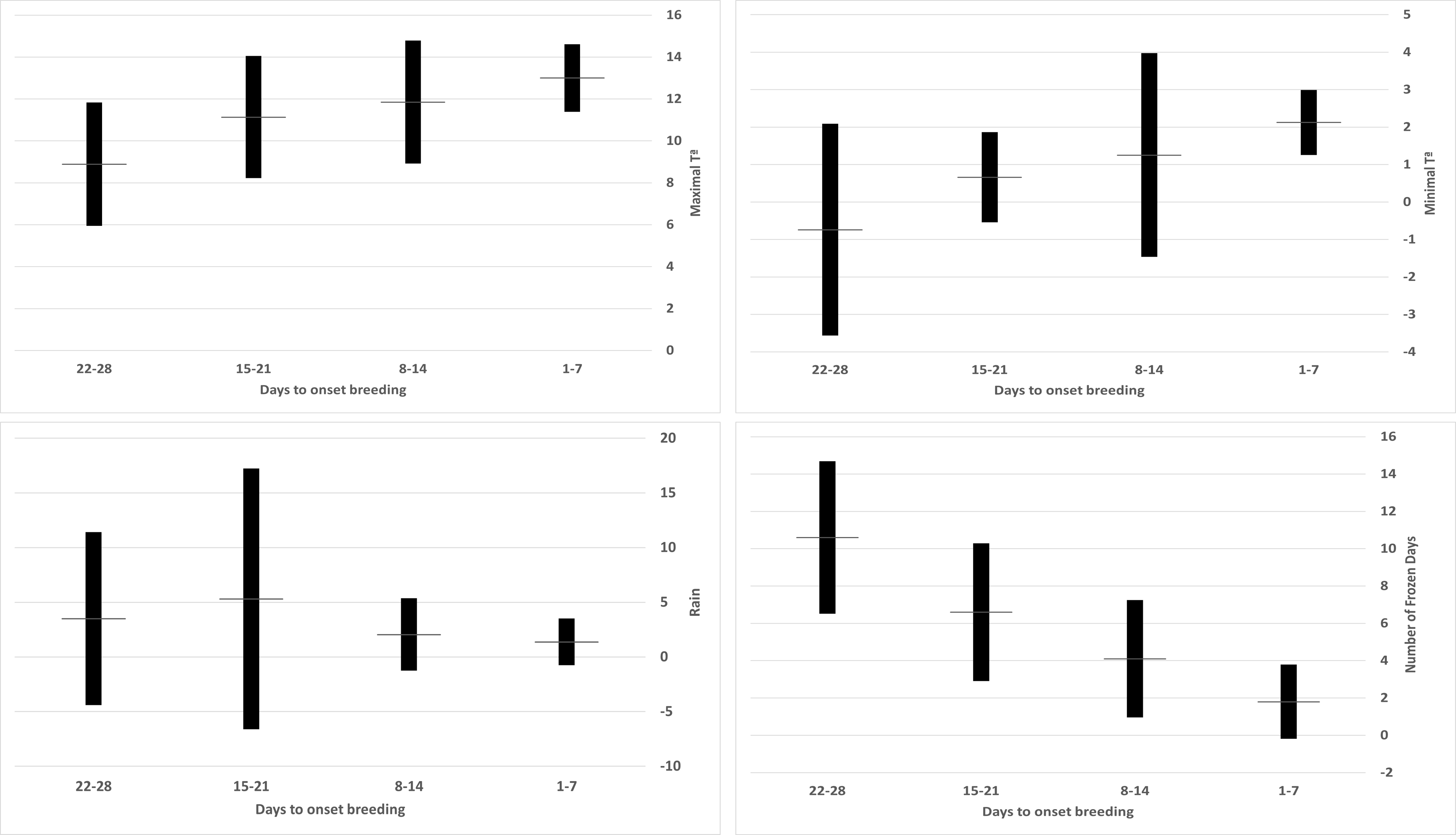

Supplement: Supplemental Information 3 [file peerj-11-14527-s003.png]

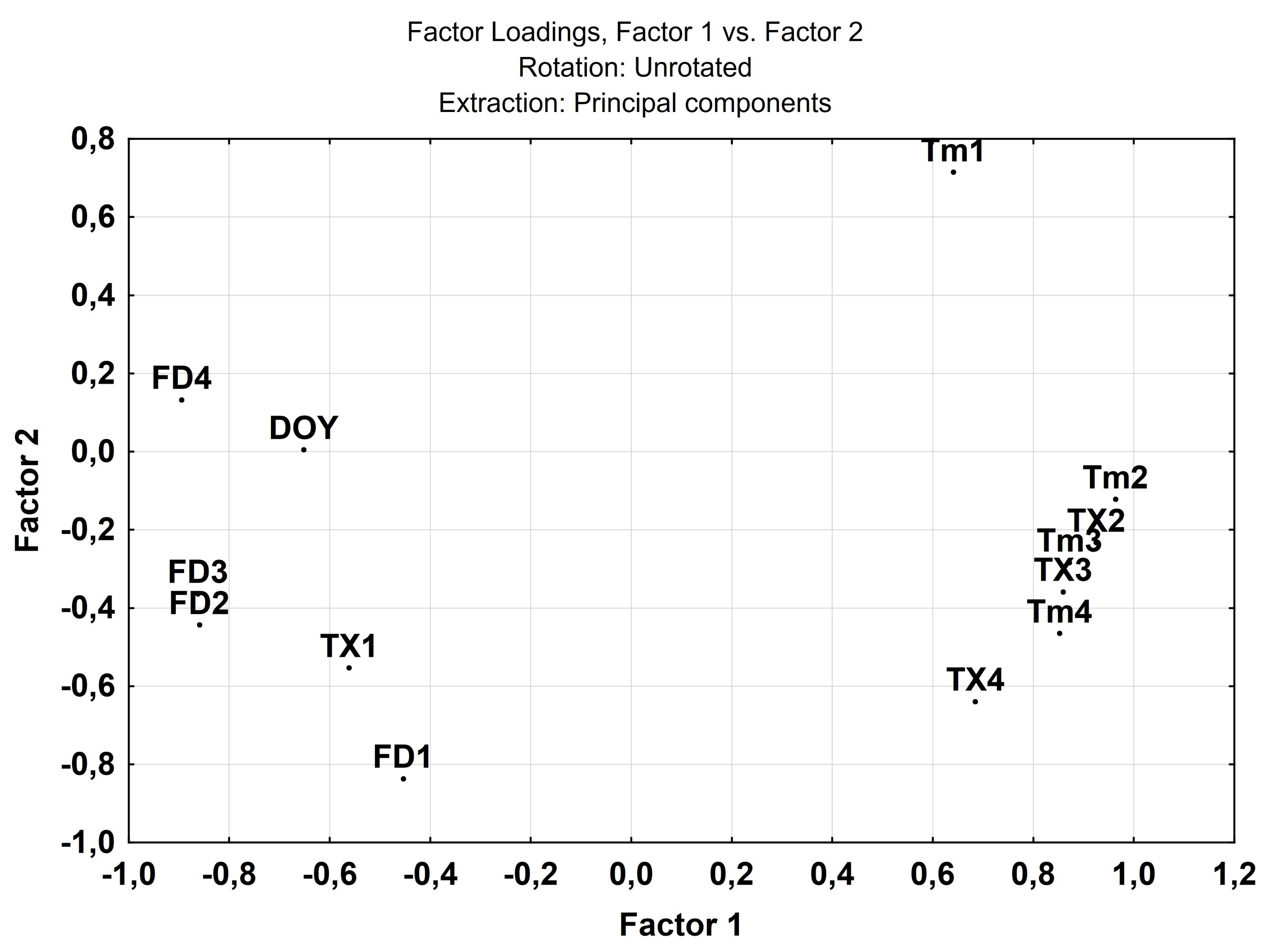

Supplement: Supplemental Information 4 [file peerj-11-14527-s004.png]

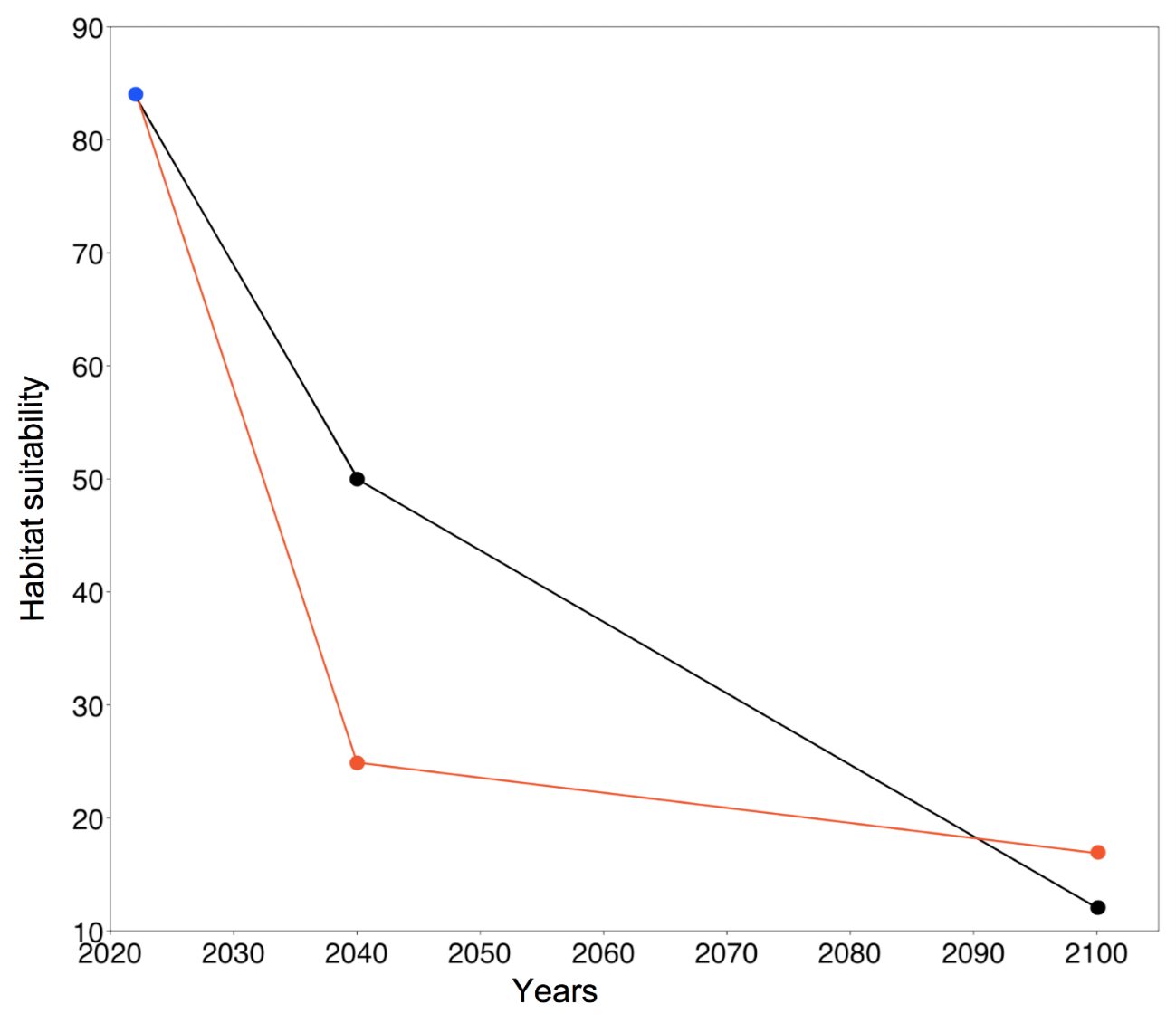

Supplement: Supplemental Information 5 — Points are the number of grids where the probability of species occurrence is ≥ 0.5. [file peerj-11-14527-s005.png]

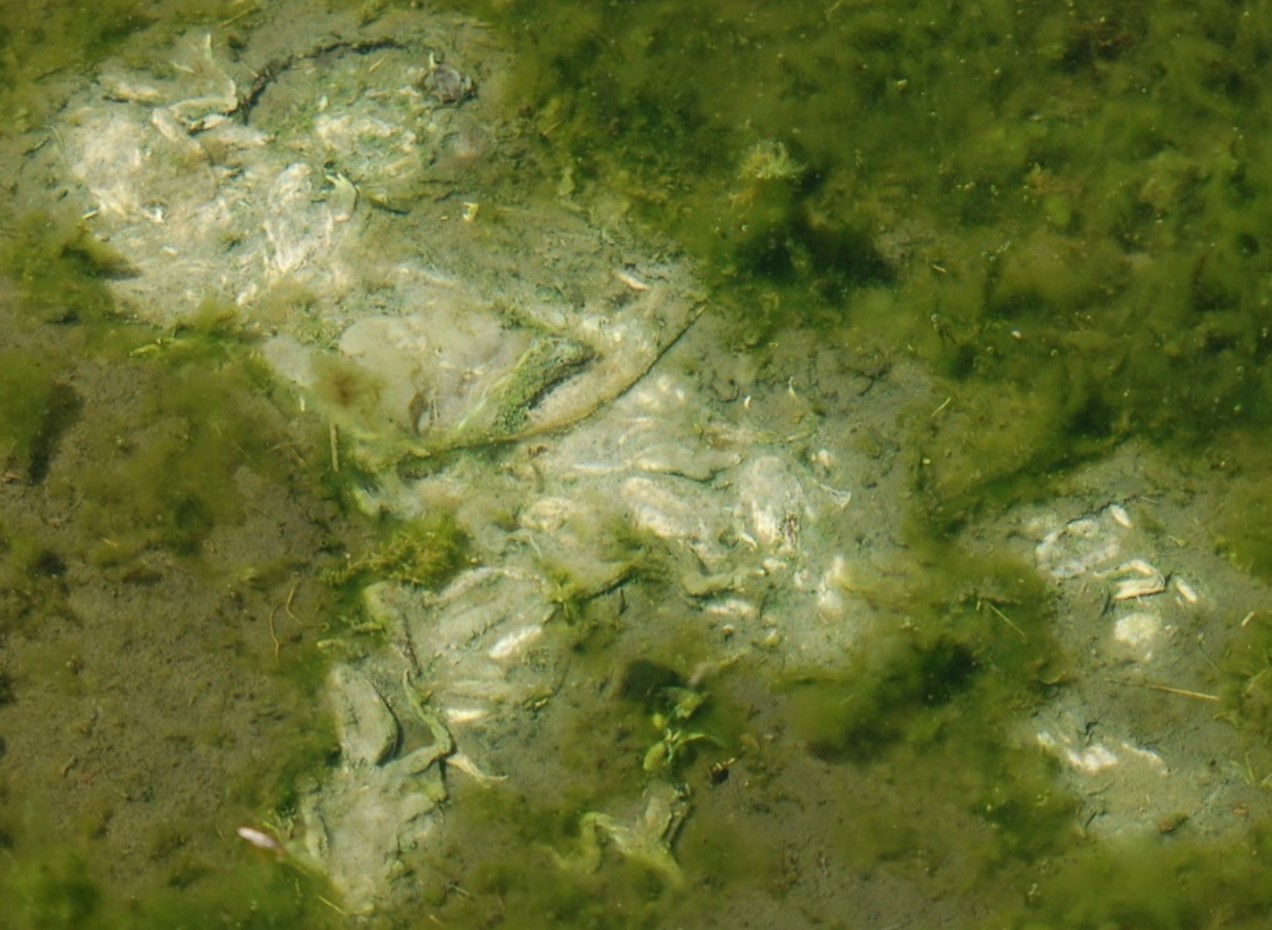

Supplement: Supplemental Information 6 — Photo credit: Albert Montori [file peerj-11-14527-s006.jpg]
